# Gas Separation by Mixed Matrix Membranes with Porous Organic Polymer Inclusions within *o*-Hydroxypolyamides Containing *m*-Terphenyl Moieties

**DOI:** 10.3390/polym13060931

**Published:** 2021-03-18

**Authors:** Cenit Soto, Edwin S. Torres-Cuevas, Alfonso González-Ortega, Laura Palacio, Ángel E. Lozano, Benny D. Freeman, Pedro Prádanos, Antonio Hernández

**Affiliations:** 1 Surfaces and Porous Materials (SMAP), Associated Research Unit to CSIC, Faculty of Science, University of Valladolid, Paseo Belén 7, 47011 Valladolid, Spain; marveliacenit.soto@uva.es (C.S.); laura.palacio@uva.es (L.P.); lozano@ictp.csic.es (Á.E.L.); 2 Institute of Sustainable Processes (ISP), Dr. Mergelina s/n, 47011 Valladolid, Spain; 3 McKetta Department of Chemical Engineering, The University of Texas at Austin, Austin, TX 78712, USA; edwinstorres@utexas.edu (E.S.T.-C.); freeman@che.utexas.edu (B.D.F.); 4 Department of Organic Chemistry, School of Sciences, Faculty of Sceince, University of Valladolid, Paseo Belén 7, 47011 Valladolid, Spain; alfonso.gonzalez.ortega@uva.es; 5 Institute for Polymer Science and Technology (ICTP-CSIC), Juan de la Cierva 3, 28006 Madrid, Spain; 6 IU CINQUIMA, University of Valladolid, Paseo Belén 5, 47011 Valladolid, Spain

**Keywords:** hydrogen separation, mixed matrix membranes, porous polymer networks, thermal rearrangement

## Abstract

A hydroxypolyamide (HPA) manufactured from 2,2-bis(3-amino-4-hydroxy phenyl)-hexafluoropropane (APAF) diamine and 5′-terbutyl-*m*-terphenyl-4,4′′-dicarboxylic acid chloride (tBTpCl), and a copolyimide produced by stochiometric copolymerization of APAF and 4,4′-(hexafluoroisopropylidene) diamine (6FpDA), using the same diacid chloride, were obtained and used as polymeric matrixes in mixed matrix membranes (MMMs) loaded with 20% (*w*/*w*) of two porous polymer networks (triptycene-isatin, PPN-1, and triptycene-trifluoroacetophenone, PPN-2). These MMMs, and also the thermally rearranged membranes (TR-MMMs) that underwent a thermal treatment process to convert the o-hydroxypolyamide moieties to polybenzoxazole ones, were characterized, and their gas separation properties evaluated for H_2_, N_2_, O_2_, CH_4_, and CO_2_. Both TR process and the addition of PPN increased permeability with minor decreases in selectivity for all gases tested. Excellent results were obtained, in terms of the permeability versus selectivity compromise, for H_2_/CH_4_ and H_2_/N_2_ separations with membranes approaching the 2008 Robeson’s trade-off line. The best gas separation properties were obtained when PPN-2 was used. Finally, gas permeation was characterized in terms of chain intersegmental distance and fraction of free volume of the membrane along with the kinetic diameters of the permeated gases. The intersegmental distance increased after TR and/or the addition of PPN-2. Permeability followed an exponential dependence with free volume and a quadratic function of the kinetic diameter of the gas.

## 1. Introduction

Today, renewable energy systems are attracting an increasing interest due to the ever-growing energy demand at a global scale, decreasing prices, and the urgent need to mitigate climate change [1]. In this context, hydrogen (H_2_) could play a key role in the energy sector since it represents a clean and cost-effective gaseous energy vector with a high specific energy content [2] that can substitute fossil fuels and thus reduce CO_2_ emissions [3,4,5]. For instance, H_2_ could replace natural gas during electricity generation in power plants or fossil fuels in transportation [6]. However, according to the most recent report of the International Energy Agency, fossil fuels are the main source of H_2_ production, which is responsible for at least 830 million tons of CO_2_ emissions per year. Indeed, H_2_ production accounted for 6% of global natural gas and 2% of global coal demand [3].

In this regard, sustainable H_2_ production from a renewable source is needed to reduce the consumption of fossil fuels and their associated CO_2_ footprint [2]. In recent years, a large research effort has been deployed to develop green technologies to generate renewable H_2_ [7]. Dark fermentation from biomass or water electrolysis using the surplus of renewable electricity rank among the most investigated technologies to generate green H_2_ [8]. On the other hand, pressure swing adsorption (PSA), cryogenic separation, and membrane separation represent the most implemented technologies for H_2_ purification [9]. According to Luo et al. [6], membrane separation is considered an energy-efficient and sustainable alternative since it does not require the regeneration of the adsorption materials or a high energy demand to decrease the temperature of the gas mixture below −78 °C.

Mixed matrix membranes (MMMs) have recently experienced the most significant advances in membrane technology for hydrogen purification [10]. MMMs consist of a mixture of organic or inorganic porous materials as dispersed phases (namely additive or filler) into a polymeric matrix as a continuous phase [10,11,12,13]. Well-designed MMMs benefit from the potential synergy between the polymeric matrix and fillers, which enhances the properties of MMMs compared to the pure polymer exhibiting superior performance in terms of the permeability-selectivity compromise [14,15]. Moreover, some polymer matrixes employed in MMMs can eventually undergo thermal transposition processes such as thermal rearrangement (TR) at high temperatures [1,16,17], which could further increase gas permeabilities. For instance, TR polymers originated from the thermal conversion of poly(*o*-hydroxyamide)s to polybenzoxazole (PBO) structures showed outstanding gas transport properties for the separation of CO_2_/CH_4_ mixtures [6,18].

The manufacture of high-performance MMMs depends on the selection of the appropriate filler to prevent the formation of non-selective voids caused [12,19]. In this sense, metal-organic frameworks (MOFs) with high surface area and porosity [20], porous aromatic frameworks (PAFs) with a large surface area and high thermal stability [21], and hypercrosslinked polymers (HCPs) with high CO_2_ adsorption [22] have been successfully used as fillers for gas separation. Recently, novel materials based on porous polymer networks (PPNs) have been used as fillers in order to prepare MMMs with promising gas permeabilities [23]. In addition, these later materials exhibit outstanding chemical and thermal properties and excellent CO_2_ adsorption capacities [24]. Despite the potential of these MMMs, there is limited knowledge of the performance of thermally rearranged membranes (TR-MMMs) for gas separation applications.

This work aimed at manufacturing and testing novel polymeric and copolymeric matrixes (derived from poly(*o*-hydroxypolyamide)s having 5′-tert-butyl-*m*-terphenyl moieties and hexafluoropropyl groups) able to undergo thermal rearrangement to TR-PBOs and also to obtain TR-MMMs with favorable gas transport properties for hydrogen separation using PPN as a filler (PPNs formed from isatin and triptycene, PPN-1, or from trifluoroacetophenone and triptycene, PPN-2). Polymers containing m-terphenyl groups typically show excellent thermal stability and high glass transition temperature [25,26]. However, these polymers showed low processability when the *m*-terphenylene derivative was not substituted (for instance, 5′-H-*m*-terphenyl derivatives) [25]. After attachment of a t-butyl group in the middle aromatic ring, the solubility improved a lot, and the gas separation properties were much better [25,27,28]. Thus, it was considered that the use of m-terphenyl units, having bulky groups, in polyamides and *o*-hydroxypolyamides should produce important improvements in the features of the materials derived from them. The PPNs herein used have superb chemical and thermal stability (higher than 450 °C) and high BET (Brunauer-Emmett-Teller) surfaces (around 650 m^2^/g [24]), and they could bear the temperatures employed for carrying out the thermal rearrangement process.

## 2. Materials and Methods

### 2.1. Materials

Anhydrous dimethyl acetamide (DMAc, 99%), pyridine (Py), dimethyl amino pyridine (DMAP), trimethylsilylchloride (TMSC, >98%), N, N dimethyl formamide (DMF), 3-methoxycarbonyl-phenylboronic acid, and tetrahydrofuran (THF, 99%) were purchased from Sigma-Aldrich (Sigma-Aldrich, St. Louis, MO, USA) and used as received. Sodium hydroxide (NaOH), sulfuric acid (H_2_SO_4_), tetrakis(triphenylphosphine) palladium(0) (Pd(PPh_3_)_4_), potassium carbonate (K_2_CO_3_), hydrochloric acid (HCl), and thionyl chloride (SOCl_2_) were obtained from Scharlau (Scharlab, Barcelona, Spain). The diacid 5′-terbuthyl-*m*-terphenyl-4,4′′-dicarboxylic acid (tBTpDA) was synthesized following a procedure previously reported [29] and subsequently was converted to the monomer 5′-tertbutyl-*m*-terphenyl-4,4′′-dichloride acid (tBTpCl). 2,2-bis(3-amino-4-hydroxy phenyl)-hexafluoropropane (APAF) was purchased from Apollo Scientific (Apollo Scientific, Stockport, Cheshire, U.K.) and purified by sublimation at 220–225 °C before use. 4,4′-(hexafluoroisopropylidene) dianiline (6FpDA) was also purchased from Apollo Scientific and purified by sublimation at 180 °C before use.

APAF and tBTpCl were employed as monomers to synthesize the homo-*o*-hydroxypolyamide tBTpCl-APAF (HPA). Additionally, a copolyamide (HPA-PA) was prepared by mixing APAF with 6FpDA (at a 1/1 mol/mol ratio) with tBTpCl. For comparison’s sake, a non-TR-able polyamide without o-hydroxy groups (PA) was prepared from the monomers 6FpDA and tBTpCl. The synthesis of HPA, PA, and HPA-PA were carried out as described elsewhere [30], and it is outlined below.

### 2.2. Monomers Synthesis

#### 2.2.1. Synthesis of 4-Carboxy-Phenylboronic Acid

**Figure 1:**
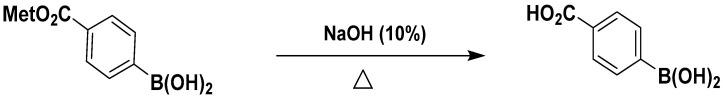
Synthesis of 4-carboxy-phenylboronic acid via saponification reaction.

A saponification reaction was used to convert the ester group of a commercial boronic acid to carboxylic acid (Figure 1). Thus, the 4-carboxy-phenylboronic acid was initially synthetized by the following procedure: In a round-bottomed flask, 113.91 mmol of 4-methoxycarbonyl-phenylboronic acid and 200 mL of a NaOH aqueous solution (10% *w/w*) were added. The solution was reflux-heated for 30 min. The resulting reaction was cooled down before filtering, and H_2_SO_4_ was added to adjust pH to 1. Due to the exothermic nature of the reaction, the solution was kept in an ice-water bath. The resulting precipitate was kept at 4 °C overnight. The precipitate was filtered, washed three times with distilled water, and allowed to dry at room temperature.

#### 2.2.2. Synthesis of (5′-Terbuthyl-m-terphenyl-4,4′′-dicarboxylic acid (p))

**Figure 2:**
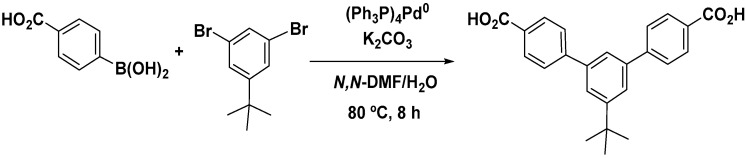
Suzuki–Miyakura synthesis of 5′-tert-butyl-*m*-terphenyl-4,4′′-dicarboxylic acid.

The Suzuki–Miyaura [31] reaction was used in order to obtain a series of derivatives of m-terphenyl-4,4′′ dicarboxylic acid, having in the 5′ position a tert-butyl group (Figure 2). This reaction involved the formation of a C-C bond through a boronic derivative (4-carboxy-phenylboronic acid) and an organohalogenide (1,3-dibromo-5-tert-butyl-benzene) in the presence of a base (K_2_CO_3_) and a palladium (0) catalyst. The synthetic procedure is outlined below.

A total of 12.5 mmol (3.64 g) of 1,3-dibromo-5-tert-buty-lbencene, 30.2 mmol (5 g) of 4-carboxy-phenylboronic acid, 1.2 mmol (1.36 g) of (Ph3P)4Pd(0), and 360 mL of DMF (previously deoxygenated) were added in a round-bottomed flask, according to a procedure adapted from Liao and Hsieh [32]. Deoxygenation using N_2_ (strictly inert atmosphere) was carried out before adding 79.8 mL of 3.2 M potassium carbonate solution to avoid the degradation of the catalyzer. The reaction was maintained at 80 °C for 8 h under magnetic stirring. Afterward, the reaction was transferred to an Erlenmeyer flask and cooled with ice before adding HCl until at pH 1. The formed suspension was then left overnight at 4 °C. The diacid compound was then separated as a solid by filtration and dissolved in 35 mL of NaOH (2 M). HCl (50:50) was added to adjust the pH to 1 to obtain a solid precipitate, which was filtered again and thoroughly washed with water and dried before rinsing with warm toluene. The resulting solid was allowed to dry at room temperature, thus obtaining tBTpDA with 88% yield.

#### 2.2.3. Synthesis of tBTpCl Dichloride

**Figure 3:**
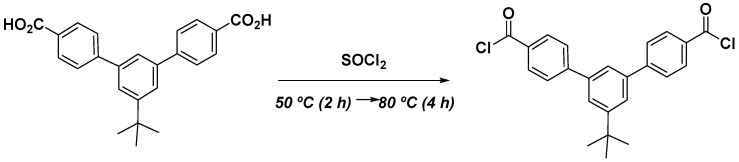
Synthesis of 5′-tert-butyl-*m*-terphenyl-4,4′′-dichloride acid (tBTpCl).

The diacid previously synthetized was added into a round-bottomed flask equipped with a reflux condenser and magnetically stirred, along with SOCl_2_ and 5 drops of DMF under a N_2_ atmosphere, following the procedure described by Smith et al. [30]. The mixture was maintained at 50 °C for 4 h and at 80 °C for 2 h and afterward allowed to cool down to room temperature (Figure 3).

Subsequently, the reflux condenser was substituted by distillation equipment to eliminate the excess of SOCl_2_, and a small amount of anhydrous toluene was added before distillation. The distillation process was initially carried out under vacuum at ambient temperature under a N_2_ atmosphere. To assure that SOCl_2_ was eliminated, anhydrous toluene was again added before increasing the temperature to 70 °C during the stripping-off process. Finally, anhydrous toluene was added before overnight cooling at 4 °C to crystallization. Finally, the liquid residue was eliminated under vacuum at 4 °C. tBTpCl was kept under a N_2_ atmosphere blanket to avoid hydration.

### 2.3. Polymers Synthesis

The procedure for the synthesis of the polymers (tBTpCl-APAF (HPA), tBTpCl-6FpDA-APAF (HPA-PA), and tBTpCl-6FpDA, (PA)) was performed by the polycondensation reaction employing the in situ silylation methodology [33,34,35,36,37]. As an example, the synthesis of the o-hydroxy homopolyamide (HPA) is described in the following paragraphs.

In a 100 mL three-necked flask equipped with a mechanical stirrer under a constant N_2_ supply, 2.0 g (0.0055 mmol) of APAF was added and dissolved in 10 mL of N,N-dimethylacetamide (DMAc). The mixture was stirred at room temperature until the complete solubilization of the solid. Then, the solution was cooled by immersing the flask into an ice bath to reach 0 °C, and 2.77 mL of TMSC were dropwise added, followed by 1.76 mL of Py. The mixture was maintained at 0 °C and stirred for 10–15 min to ensure the formation of the silylated diamine [38]. Then, 2.25 g (0.0055 mmol) tBTpCl dichloride was poured into the flask and rinsed with 2 mL of DMAc. Finally, 0.267 g (2.18 mmol) of DMAP and 32 mL of DMAc were added. The reaction was stirred for 24 h at room temperature to complete the polymerization reaction. The resulting polymers were obtained as fiber-like shape and then washed with water, a 1/1 ethanol/water mixture, and collected by filtration. It was heated under vacuum at 100 °C for 24 h to obtain a dry polymer.

### 2.4. Casting Of Polymer Films

#### 2.4.1. Films of Polymer Matrixes

To prepare the membranes, the synthesized polymers were put in 10% (*w*/*v*) THF solutions and maintained under mechanical stirring until its complete dissolution. Before casting onto a glass plate, the solution was filtered through a 4.5 µm PTFE membrane filter to remove impurities. Part of the solvent was evaporated at room temperature overnight. The remaining solvent was slowly dried in a vacuum oven (Thermo Fisher Scientific Inc, Waltham, MA, USA) with the following protocol: 60 °C for 2 h and 80 °C for 2 h without vacuum, 100 °C for 2 h, 120 °C for 1 h, and finally 180 °C for 12 h under vacuum. The membranes manufactured presented thickness between 40–60 µm.

#### 2.4.2. Preparation of Mixed Matrix Membranes

Mixed matrix membrane (MMMs) were prepared as described elsewhere [17,23]. The synthesized polymeric matrixes above were mixed with porous polymer networks (PPNs) synthetized according to Lopez-Iglesias et al. [24], which were used as fillers of the polymer matrix. A total of 1200 mg of each polymeric matrix was separately dissolved in 10% THF (*w*/*v*). The filler (20% (*w*) of the total mass) was dissolved in 10% (*w*/*v*) THF; it was sonicated for 20 min at 30% of maximum amplitude (40 cycles of 20 s sonication followed by 10 s cooling-down) before being mixed with the polymer matrix. This mixture was spread out onto a glass casting plate and subjected to the same heat treatment that was used for making the polymer matrix membrane to remove the solvent. The resulting MMMs were: MMM-HPA, MMM-HPA-PA, and MMM-PA, as described in supporting information (Table S1). In this study, two PPNs were tested (triptycene-isatin, namely PPN-1 and triptycene-trifluoroacetophenone (TFAP), namely PPN-2) [24] and selected as suitable candidates. Nevertheless, after a preliminary evaluation, PPN-2 was chosen for the rest of the study because its corresponding MMMs showed better gas separation properties (as shown in Figure supporting information section, Table S2, and Figure S1).

### 2.5. Thermal Rearrangement

**Figure 4:**
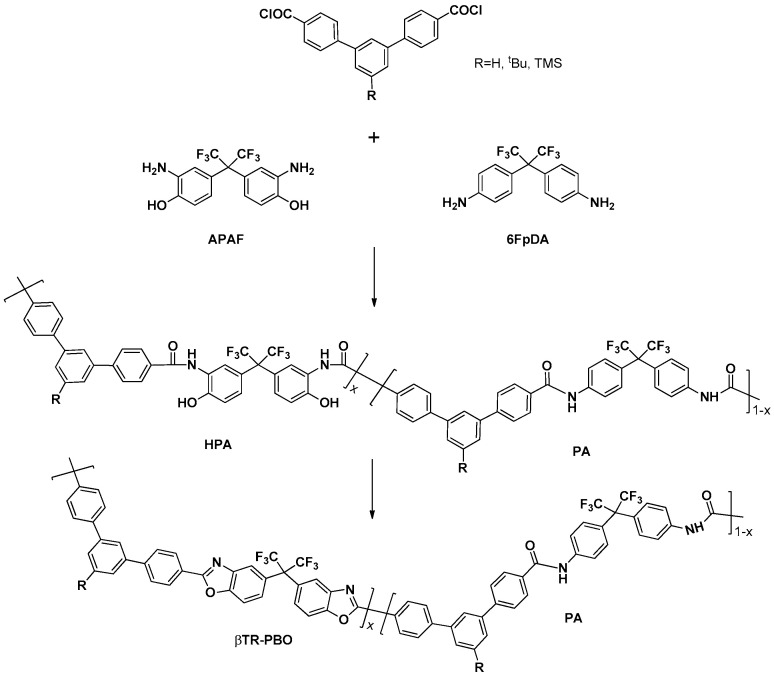
Scheme of the synthesis of HPA-PA and conversion to its corresponding TR-HPA-PA.

The resulting membranes were subjected to a thermal rearrangement (TR) process to obtain β-TR-PBO (polybenzoxazole) membranes in a carbolite split-tube furnace equipped with a quartz tube and using an ultra-high-purity nitrogen flow rate at 900 mL/min, following the Sanders et al. procedure [39]. Figure 4 displays the TR process for the conversion of HPA and HPA-PA to TR-HPA and TR-HPA-PA. Note that PA does not experience any thermal rearrangement. The samples were placed between two ceramic plates separated by stainless steel washers, and they were initially heated at 5 °C/min up to 250 °C and held for 15 min. The second ramp (5 °C/min) increased the temperature up to 375 °C, which was held for 15 min. Finally, samples were cooled to ambient temperature at 10 °C/min and maintained under a nitrogen flow.

### 2.6. Characterization

#### 2.6.1. Polymer Characterization

Weight-average molecular weights (Mw) and number-average molecular weights (Mn) of the polymers synthesized were determined by gel permeation chromatography (GPC) using a Tosoh Ecosec HLC-8320GPC (Tosoh, Tokyo, Japan) device. Samples were prepared by dissolving 0.5 mg of each polymer in 2 mL of THF and filtered through a 0.45 µm filter.

^1^H and ^13^C nuclear magnetic resonance (NMR) spectra were performed using a Varian AV Agilent (Varian, Palo Alto, CA, USA) working at 400 MHz and 100 MHz. The NMR samples were prepared using deuterated dimethyl sulfoxide (DMSO-*d*6) to dissolve the polymer fibers.

Polymer solubility was determined by placing ~10 mg of the polymer in 1 mL of the target solvent (N,N-dimethylacetamide, DMAc, N-methylpyrrolidone, NMP, tetrahydrofuane, THF, chloroform, CHCl_3_, m-cresol, acetone, ethanol, N,N-dimethylformide, DMF) in solubility tubes until its total dilution. If the polymer was not soluble at room temperature, the solution was heated to the boiling point of the solvent, and its solubility checked.

#### 2.6.2. Thermogravimetric Analysis

Thermal rearrangement of the membranes (mass loss and decomposition products) was performed via thermogravimetric analysis (TGA) using a TA Instruments (TA Instruments, New Castle, DE, USA) Q500 thermogravimetric analyzer in 5 mg samples. Ultra-high-purity nitrogen at a flow rate of 40 mL/min in the balance and 50 mL/min in the sample was used. The temperature ramp was set at 10 °C/min up to 800 °C.

#### 2.6.3. DSC

To monitor the glass transition temperatures (Tg), differential scanning calorimetry (DSC) was carried out in a TA Instruments DSC Q-20 Analyzer (TA Instruments-Water Corp., Milford, MA, USA). DSC analyses for TR polymers were carried out at a heating rate of 20 °C/min up to 360 °C. In all cases, the experiments were performed under a N_2_ atmosphere using 6–10 mg of membranes in gas-tight aluminum containers. The glass transition temperature (Tg) was determined in the second heating cycle from the middle point of the resulting slopes.

#### 2.6.4. Fourier Transform Infrared Spectroscopy

The conversion of the resulting membranes to TR-PBO membranes was monitored via attenuated total reflectance-Fourier transform infrared (ATR-FTIR) using a PerkinElmer Spectrum One FT-IR (PerkinElmer, Waltham, MA, USA) coupled with a universal attenuated total reflection (ATR) diamond-tipped sampling module following the band’s intensity.

#### 2.6.5. Density Measurements

The fractional free volume (FFV) is defined as:

(1) $$FFV=\frac{V-V_{0}}{V}$$

Here V is the total specific volume and $V_{0}$ is the specific skeletal volume of MMM. The skeletal volume for HPA, PPN, and MMMs can be evaluated from their van der Waals volumes because $V_{0}\approx 1.3\;V_{w}$. Actually, $V_{w}^{HPA}$ and $V_{w}^{PPN}$ can be calculated by molecular modeling using the Materials Studio software (BioVia, San Diego, CA, USA). To evaluate $V_{w}^{MMM}$, we can use:

(2) $$V_{w}^{MMM}=\phi V_{w}^{PPN}+(1-\phi )V_{w}^{HPA}$$

This equation correlates specific van der Waals volumes in terms of ϕ, the fraction of filler (PPN). Once $V_{w}^{MMM}$ is known, we can obtain $V_{0}^{MMM}$.

The total specific volumes can be obtained from the corresponding densities:

(3) $$\begin{array}{l} \rho^{MMM}=1/V^{MMM}\\ \rho^{HPA}=1/V^{HPA} \end{array}$$

*V^PPN^* can be obtained from Equation (2):

(4) $$V^{PPN}=\frac{1}{\phi }\left[V^{MMM}-(1-\phi )V^{HPA}\right]$$

Densities were measured by following the Archimedes principle in a CP225 Analytical Balance from Sartorius (Sartorius, Göttingen, Germany) equipped with a density measurement kit. The samples were weighed in air and into high pure isooctane at room temperature. The average density from seven samples was obtained as:

(5) $$\rho =\rho_{C_{8}H_{18}}\frac{W_{air}}{W_{air}-W_{C_{8}H_{18}}}$$

where $\rho_{C_{8}H_{18}}$ corresponds to the isooctane’s density, W_air_ to the weight of the sample, and $W_{C_{8}H_{18}}$ stands for the weight of the sample when submerged in isooctane. Finally, Equation (1) allows the determination of FFV.

#### 2.6.6. WAXS

The membranes were tested via wide-angle X-ray scattering (WAXS) at room temperature using a Bruker (Bruker, Billerica, MA, USA) D8 Discover A25 advanced diffractometer equipped with a Goebel mirror. The LynxEye detector was operated at a speed of 0.5 s with a step scanning mode ranging from 5° to 70° and a 2θ step of 0.020°. A Cu Kα (λ = 1.542 Å) radiation source in a ceramic tube was used.

#### 2.6.7. Mechanical Properties

Mechanical properties of the polymeric matrixes, MMMs, and their corresponding TR membranes were determined using a Shimadzu Autograph AGS-X 500N tensile testing instrument (Shimadzu, Kyoto, Japan). The tensile test was set to 1 mm/min to assess the crosshead speed. Samples were cut using a microtensile dog bone-shaped die before heat treatment. The gauge length and width (~22 mm and 5 mm, respectively) were measured by a digital scanner using ImageJ software to measure the average width and gauge length. Membrane thicknesses were measured by a Mitutoyo digital caliper (Mitutoyo, Kawasaki, Kanagawa, Japan) of ±1 µm resolution. Five replicate measurements were carried out for each membrane tested.

#### 2.6.8. Gas Transport: Permeability and Selectivity

Membranes with a uniform thickness were placed on a support of brass disks (using epoxy as adhesive and protected with glass fiber filter paper) to determine gas permeability. This epoxy was dried at room temperature for 3 h followed by 3 h at 60 °C before use. Single gas (except H_2_) permeability was determined using a constant-volume apparatus, as described elsewhere [17]. H_2_ permeability was measured at the University of Texas at Austin facilities using a different but similar constant-volume apparatus [40]. All permeability measurements were performed at 35 °C and an upstream pressure of 3 bar.

The dry sample was placed onto the permeation cell using the constant-volume variable-pressure method. Gas permeability (cm^3^ (STP) cm/ (cm^2^ s cmHg)) was determined by:

(6) $$P=\frac{V_{d}l}{p_{2}ART}\left[{\left(\frac{dp_{1}}{dt}\right)}_{ss}-{\left(\frac{dp_{1}}{dt}\right)}_{leak}\right]$$

where *V_d_* is the downstream volume (cm^3^) of a permeation system, l is the membrane thickness (cm), *p*_2_ is the upstream pressure (cmHg), *A* is the area available for the gas transport (cm^2^), the universal gas constant *R* is 0.278 cmHg cm^3^/(cm^3^(STP) K), T is the absolute temperature (K), and (d*p*_1_/d*t*)leak is the steady-state rates of pressure rise (cmHg/s) in the downstream volume. Gas selectivity as:

(7) $$\alpha_{A/B}=\;\frac{P_{1}}{P_{2}}$$

Samples were kept under vacuum overnight at 35 °C before testing to remove any adsorbed gas. The permeabilities of He, H_2_, O_2_, N_2_, CH_4_, and CO_2_ (99.999% purity) supplied by Airgas (Airgas, Radnor, Pennsylvania, USA) were measured at 3 bars at 35 °C. He permeability was measured at 1, 2, and 3 bars to detect pinholes through the membranes prior to any ulterior membrane testing. All gases were tested before CO_2_ measurement to prevent membrane plasticization. A vacuum was implemented for at least 20-fold the time lag before measuring the permeability for a new gas.

## 3. Results and Discussion

### 3.1. Chemical Properties

#### 3.1.1. Characterization of Polymer Matrixes

The average molecular weight (Mw) of each polymer was determined to be 163.1, 158.4, and 84.6 kDa for HPA, PA, and HPA-PA, respectively.

The solubility of the polymers herein synthesized was carried out in different solvents. All polymers were soluble in a common organic solvent such as THF and in polar aprotic solvents such as DMAc, NMP, and DMF. None of the polymers synthesized were soluble, neither in CHCl_3_ nor in ethanol.

The corresponding NMR spectra are shown in the supporting information (Figures S2–S4).

#### 3.1.2. Infrared Spectroscopy (FTIR) Measurements

**Figure 5:**
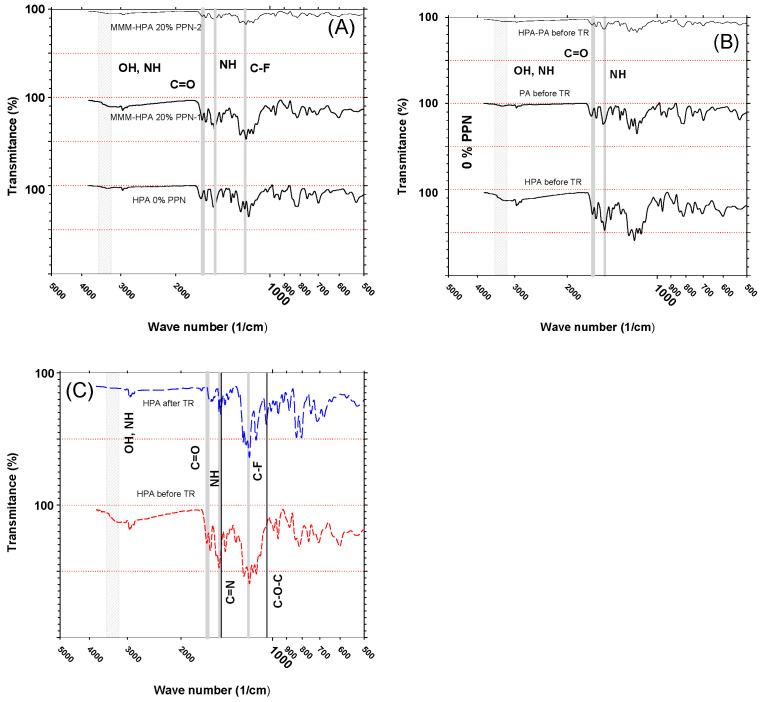
Infrared Spectroscopy (FTIR) spectra of polymeric matrixes (**A**), mixed matrix membranes (MMMs) with triptycene-isatin (PPN-1) and triptycene-trifluoroacetophenone (PPN-2) compared with the corresponding polymeric matrix before thermal rearrangement (**B**), and the polymeric matrix after thermal rearrangement (**C**).

Figure 5A shows the absorption bands for the polymeric matrixes used here before any thermal rearrangement, where an O-H vibration band (3200–3500 cm^−1^) related with the hydroxyl groups and a N-H band related with the amine group are observed. A stretching vibration band for C=O (1638 cm^−1^) and a N-H symmetric band (1497 cm^−1^) were also identified.

In Figure 5B, the stretching vibration band for the C-F group (~1200 cm^−1^) was observed, the presence of this band being typically associated with the filler of the MMMs. According to Lopez-Iglesias et al. [24], this absorption band was related to the PPN-2 filler.

The conversion of the precursor membranes to β-TR-PBO ones was confirmed by the presence of C=N stretching oxazole I and C-O-C stretching oxazole II (1475 cm^−1^ and 1043 cm^−1^, respectively) absorption bands, which are characteristics of benzoxazoles (Figure 5C).

### 3.2. Thermal Properties

#### 3.2.1. Thermogravimetric Analysis

**Figure 6:**
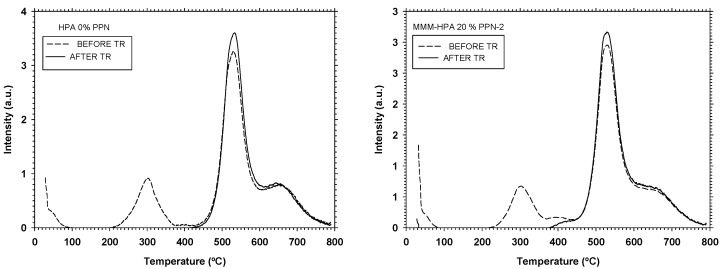
Thermogravimetric analysis (TGA) thermograms of (**left**) HPA and (**right**) MMM-HPA. Samples were heated from 50 to 800 °C at 5 °C/min under a N_2_ atmosphere.

Thermogravimetric analysis was carried out to elucidate the characteristics of the thermal conversion of HPA, HPA-PA, and their corresponding MMMs to β-TR-PBOs materials [18]. The thermal conversion occurs through a cyclization process, obtaining the polybenzoxazole after dehydration. Figure 6 displays the thermograms for HPA and MMM-HPA before and after thermal rearrangement. The corresponding thermograms for PA, MMM-PA, HPA-PA, and MMM-HPA-PA are shown in the supporting information section (Figure S5). In all cases, two common regions of weight loss for the membranes were observed. The first step (in the range from 200 to 400 °C) of weight loss was associated with the thermal rearrangement from polymer matrix moieties to benzoxazole ones, whereas the second step (well above 400 °C) was associated with thermal degradation [18,41,42,43]. Note that polymer degradation occurred above 450 °C in the case of MMMs, which ruled out any potential degradation of the PPNs due to its high thermal resistance (PPN-2 degradation occurred around 490 °C under a N_2_ atmosphere) [24].

#### 3.2.2. DSC for Glass Transition Temperatures

**Figure 7:**
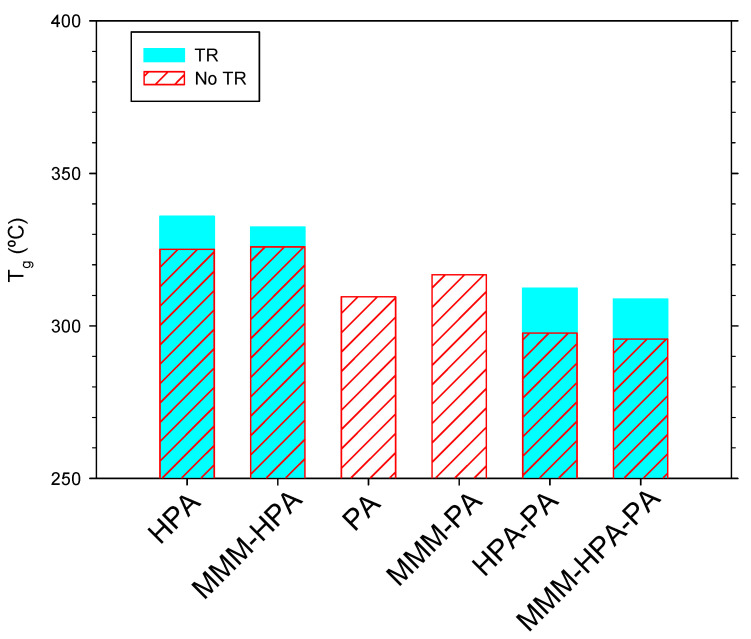
Glass transition temperatures for the membranes studied. MMMs include 20% PPN-2.

DSC spectra carried out to determine the glass transition temperatures are shown in Figure 7.

It is seen that Tg always increased after thermal rearrangement. Note that no thermal rearrangement process for the PA or MMM-PA membranes is possible. Other tendencies could be estimated from these figures, although they are small and possibly covered by the error ranges. In particular, it seems that before the treatment, addition of PPN-2 has little effect on Tg, except for PA that showed a clear increase of Tg after adding PPN-2. It appears that, in all cases, the presence of the filler decreases Tg after thermal rearrangement.

### 3.3. Mechanical Properties

Table 1 summarizes the mechanical property data for MMMs derived from PPN-2; maximum stress, elongation at break, and Young’s modulus. The elongation break was moderate (7.5–10.1%) and comparable for all matrix polymers, MMMs, and their corresponding TR-MMMs, whereas Young’s modulus decreased with the addition of PPN-2 and when subjected to thermal treatment. For instance, Young’s modulus decreased from 2.9 ± 0.2 GPa to 1.7 ± 0.1 GPa with the addition of the filler in HPA and to 1.3 ± 0.1 GPa when thermal treatment was applied. The membranes prepared from HPA polymer exhibited high maximum stress (103.3 ± 4.4 MPa). The addition of 20% PPN-2 induced a decrease in the maximum stress regardless of the membrane tested. Interestingly, thermal rearrangement entailed an increase in the maximum stress.

### 3.4. Gas Separation Properties

In a preliminary study, as shown in the supporting information section (Table S2 and Figure S1), it was observed that the MMMs and TR-MMMs derived from PPN-1 showed worse gas separation properties than those derived from PPN-2, and thus, PPN-2 was selected as the filler employed for further characterization.

The values of permeabilities of the pure gases (H_2_, N_2_, O_2_, CH_4_, and CO_2_) of the precursors, MMMs, and TR-MMMs materials, derived from PPN-2, are displayed in Table 2.

Thermal rearrangement of the HPA caused a remarkable increase in gas permeability regardless of the gas tested, with values increasing 5-, 17-, 12-, 7-, and 13.5-fold for H_2_, N_2_, O_2_, CH_4_, and CO_2_, respectively, compared to the non-TR polymeric matrix. The permeabilities of H_2_, N_2_, O_2_, CH_4_, and CO_2_ notably increased with the addition of 20% PPN-2 to the polymeric matrix by a factor of 4.0–10.2 for HPA, 2.2–2.5 for PA, 3.1–4.3 for the HPA-PA copolymer, and 2.5–2.8 for TR-HPA. Overall, the largest increase in permeability was observed for CH_4_ and CO_2_. Moreover, the permeabilities of all the gases studied are always ordered according to their kinetic diameters.

MMMs’ gas transport properties are known to be strongly determined by the morphology of the interface [13]. This morphology, in turn, could be caused, at least partially, by the fact that the polymer networks herein used contained solvent inside their pores, and the removal of this solvent from both the matrix and the filler could not be wholly effective. Despite that this could also limit the total polymer conversion to PBO, this phenomenon was not observed for TR-MMMs since the solvent was completely eliminated during the TR process, and therefore, their gas separation properties present lower variability in terms of permeability and selectivity. In any case, heat treatment might be optimized to maximize the rate of solvent removal/conversion to PBO.

It is worth noting that MMMs derived from PA exhibited the best gas separation performance for all tested gases.

The most important improvement in gas permeabilities was recorded for TR-MMMs, particularly for those manufactured from HPA. Several investigations have attributed the effect of the filler on permeability (increase) and on selectivity (slight decrease) to the high permeability provided by the poor packing of the polymer chains in MMMs [17,39,44,45]. Likewise, Park et al. reported that diffusivity significantly impacted gas transport in TR membranes as a result of their high free volume fraction, which is typically associated with microporous structures appearing as a consequence of the subsequent rearrangement [46]. The slight decrease of selectivity linked to the addition of the filler has been attributed to poor polymer-filler adhesion [47].

**Figure 8:**
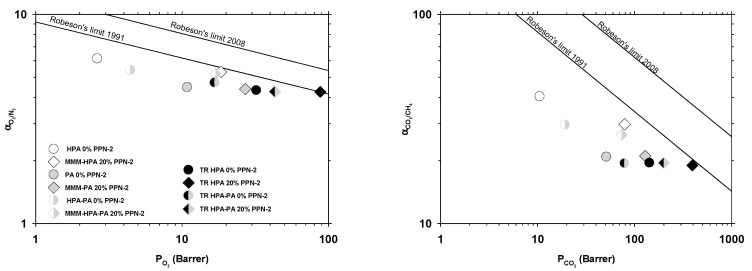
Permeability-selectivity Robeson’s plot for the O_2_/N_2_ pair (**left**) and the CH_4_/CO_2_ pair (**right**).

The 1991 and 2008 Robeson upper bound plots [48,49] for the O_2_/N_2_ and CO_2_/CH_4_ gas pairs are shown in Figure 8. Note that some results reach the 1991 Robeson’s upper bound.

**Figure 9:**
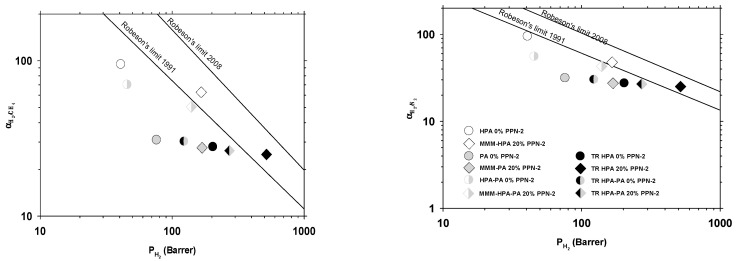
Permeability-selectivity Robeson’s plot for the H_2_/CH_4_ (**left**) and H_2_/N_2_ (**right**) gas pairs.

In Figure 9, the corresponding Robeson’s plots are shown for H_2_/CH_4_ and H_2_/N_2_. These pairs show the better permeability versus selectivity behavior with some results over the 1991 Robeson’s trade-off line and close to the 2008 line.

**Figure 10:**
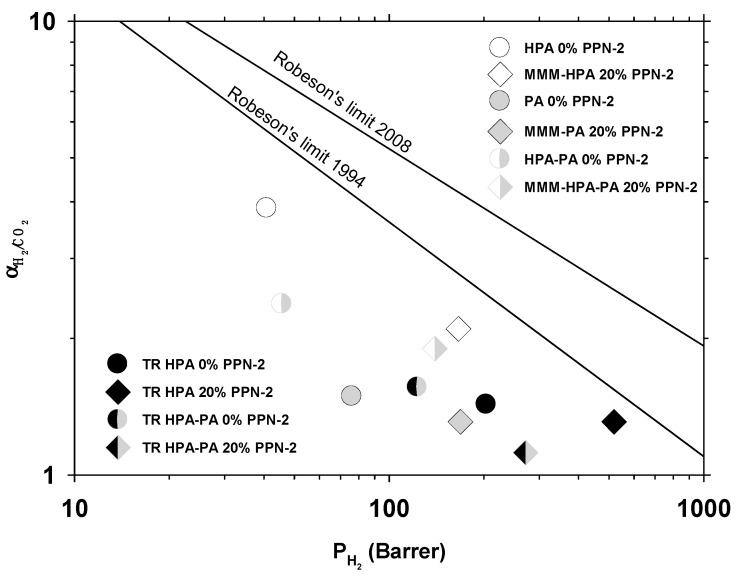
Permeability-selectivity Robeson’s plot for the H_2_/CH_4_ (**left**) and H_2_/N_2_ (**right**) gas pairs.

The slightly worse selectivity versus permeability results for the H_2_/CO_2_ pair are shown in Figure 10. Note that the earlier upper bound relationship for H_2_/CO_2_ was published in 1994 [50].

The best gas transport properties were recorded for the TR-MMMs derived from the blend of HPA, supplemented with 20% of PPN-2, which exceeded the Robeson limit 1991 for the H_2_/CH_4_ and H_2_/N_2_ gas pairs. Thus, it reached the 1991 upper bound for O_2_/N_2_ and CO_2_/CH_4_ only after thermal rearrangement. On the contrary, it does not reach the 1994 upper bound, not even after thermal rearrangement for H_2_/CO_2_. A significant increase in CO_2_ permeabilities compared to the MMMs without thermal rearrangement was observed for the TR-MMMs, which exhibited an excellent CO_2_ affinity leading to the high solubility of this gas within the polymer matrix, thus obtaining a superior gas transport [51].

### 3.5. Morphology of the MMMs

#### 3.5.1. Density and Fractional Free Volume (FFV)

Because permeability can be written as P=SD (the product of solubility *S* and diffusivity *D*) and *D* depends on the fraction of free volume, we will call f≡FFV to easy notation, as $D=Ae^{Bf}$, as shown by Thornton et al. [52]:

(8) $$P=SD=Ae^{\beta f}$$

It can be assumed that this equation holds when solubility is almost independent of FFV (*f*) or depends, like diffusivity, exponentially on *f*. Several models based on a reasonable linear dependence of the diffusion activation energy with the transversal area of the penetrant admit a quadratic dependence of β with the kinetic diameter, *d_k_* [52,53,54]:

(9) $$\beta =a+bd_{k}+cd_{k}^{2}$$

Combining Equations (8) and (9), we get:

(10) $$\ln P=\ln A+\beta f=\left[\ln A+af\right]+\left[bf\right]d_{k}+\left[cf\right]d_{k}^{2}$$

**Figure 11:**
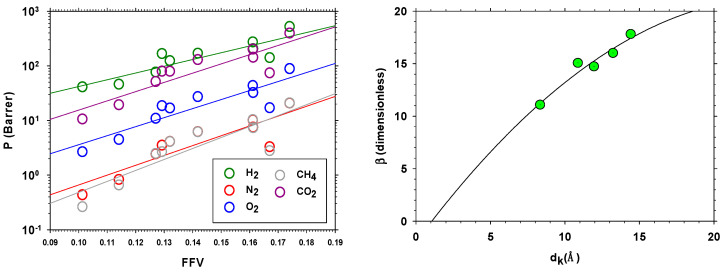
Permeability-selectivity Robeson’s plot for the H_2_/CH_4_ (**left**) and H_2_/N_2_ (**right**) gas pairs.

Figure 11 shows P versus *f* and β as a function of d_k_ for the membranes containing PPN-2, showing suitable accordance with Equations (9) and (10). Here the kinetic diameters are given by Breck [53,55].

#### 3.5.2. WAXD Intersegmental Distance

**Figure 12:**
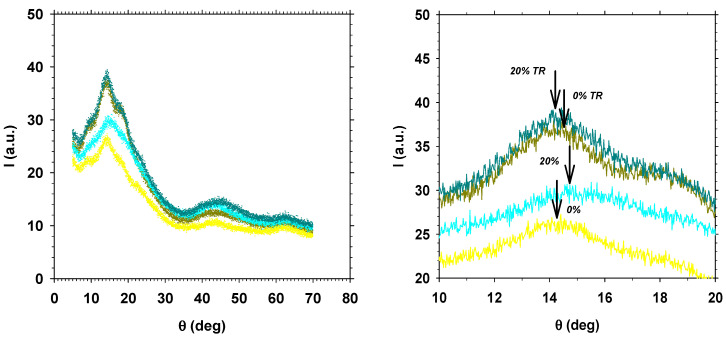
WAX spectra for HPA (**left**) and amplification of the corresponding peaks (**right**).

Figure 12 shows the WAXD spectra for HPA materials, without and with PPN-2, before and after thermal rearrangement.

**Figure 13:**
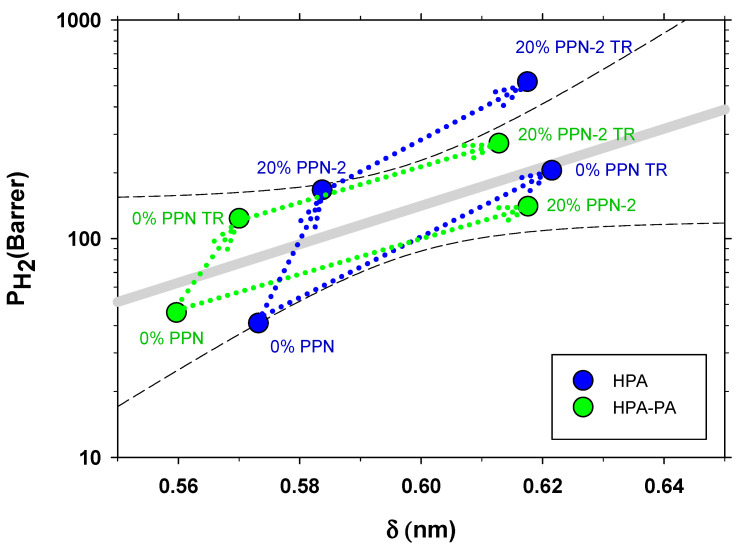
Hydrogen permeability as a function of the most probable intersegmental distance as evaluated by WAX.

In Figure 13, the permeability of hydrogen is shown as a function of the most probable intersegmental distance (δ) as evaluated by WAX. It is clearly seen there that the thermal rearrangement process and addition of PPN-2 increase the intersegmental distance and permeability. The addition of PPN-2 causes small increases in δ but causes a relatively large change in permeability. Both permeability and δ are lower for the copolymer. Note that for a similar system, we found [17] an opposite trend. Then, a decrease of δ was recorded when increasing amounts of PPN were loaded (with simultaneous increase of permeability). This was probably due to a stronger effect of the filler on the structure of the polymeric matrix, causing its compaction with the appearance of transport paths attributable to flaws and intercommunicated interstices.

## 4. Conclusions

A set of an o-hydroxypolyamide (HPA), produced by reaction of 2,2-bis(3-amino-4-hydroxyphenyl)-hexafluoropropane (APAF) diamine and 5′-tert-butyl-*m*-terphenyl-4,4′′-dicarboxylic diacid chloride (tBTpCl), a copolymer (HPA-PA) combining APAF and 6FpDA (1/1 mol/mol%) was also produced by reaction with tBTpCl, and finally a polyamide (PA) derived from with tBTpCl and 4,4′-(hexafluoroisopropylidene) (6FpDA), without *o*-hydroxy moieties, were obtained and thoroughly characterized. Mixed matrix membranes (MMMs) produced from HPA, HPA-PA, and PA employing 20% *w/w* of two porous polymer networks (PPNs) (triptycene-Isatin PPN-1 and triptycene-trifluoroacetophenone, PPN-2) were obtained. It was observed, in a preliminary gas separation study, that the best gas separation results were observed for the MMMs produced from PPN-2, and consequently, it was chosen as the only PPN load.

In addition, the polymer membranes and their corresponding MMMs were thermally treated to temperatures around 350 °C in order to produce the thermal rearrangement of the *o*-hydroxypolyamide moieties to benzoxazole ones.

All the membranes showed suitable mechanical properties able to withstand the pressures employed in gas separation applications.

Gas separation properties have been tested for H_2_, N_2_, O_2_, CH_4_, and CO_2_. Thermal rearrangement and the addition of porous polymer networks increased permeability with a slight decrease in selectivity for all gas pairs studied. Remarkably, good results have been obtained for the H_2_/CH_4_ and H_2_/N_2_ pairs. In both cases, results approached the 2008 Robeson’s limit line. The evaluation for the O_2_/N_2_ and CO_2_/CH_4_ gas pairs provided worse results even though some results reached the 1991 Robeson’s upper bound. In all cases, the HPA-PA copolymer membranes provided intermediate results in between those observed for HPA and PA membranes.

Finally, intrachain characteristic lengths (WAXD) and the fraction of free volume (FFV) of the membrane, along with their kinetic diameters, were very useful to understand permeability values. It has been shown that thermal rearrangement and/or the addition of PPN-2 increased the intersegmental distance and, consequently, permeability. The presence of PPN-2 increased the intersegmental distance slightly with a relatively high increase in permeability. Permeability has been shown to follow an exponential dependence with free volume and a quadratic function with the kinetic diameter of the gas.

## Figures and Tables

**Table 1:** Mechanical properties of HPA, PA, MMMs, and their corresponding thermally rearranged membranes (TR-MMMs).

Membrane	Maximum Stress (MPa)	Elongation at Break (%)	Young’s Modulus (GPa)
HPA	103.3 ± 4.4	9.7 ± 0.4	2.9 ± 0.2
TR-HPA	79.2 ± 9.2	9.2 ± 0.5	1.8 ± 0.3
MMM-HPA	30.5 ± 2.0	9.2 ± 0.7	1.7 ± 0.1
TR-MMM-HPA	37.7 ± 9.9	9.0 ± 0.3	1.2 ± 0.1
PA	89.8 ± 3.8	10.1 ± 0.6	2.4 ± 0.09
MMM-PA	32.2 ± 10.5	7.6 ± 2.8	1.6 ± 0.097
HPA-PA	90.2 ± 14.2	9.974 ± 0.3	2.609 ± 0.3
TR-HPA-PA	n.a.	n.a.	n.a.
MMM-HPA-PA	37.4 ± 5.2	9.648 ± 0.4	1.907 ± 0.2
TR-MMM-HPA-PA	43.96 ± 8.00	9.778 ± 0.2	1.475 ± 0.1

**Table 2:** Pure gas permeabilities using PPN-2 as filler.

Membrane	Permeabilities (Barrer ^1^)				
	H_2_	N_2_	O_2_	CH_4_	CO_2_
Non-TR materials					
HPA	40.84	0.43	2.64	0.26	10.54
MMM-HPA	166.02	3.48	18.46	2.65	79.0
PA	76.14	2.42	10.85	2.46	51.23
MMM-PA	168.65	6.15	27.02	6.12	128.7
HPA-PA	45.65	0.82	4.44	0.65	19.18
MMM-HPA-PA	139.8	3.26	16.92	2.77	73.55
TR materials					
TR-HPA	122.9	4.07	16.71	4.06	78.8
TR-MMM-HPA	271.5	10.10	43.15	10.29	200.38
TR-HPA-PA	203.7	7.43	32.17	7.29	142.1
TR-MMM-HPA-PA	518.6	20.65	87.97	20.80	394.16

^1^ Barrer = 10^−10^ cm^3^ (STP) cm/cm^2^ s cmHg or, in SI units, 1 Barrer = 3.35 × 10^−16^ (mol m)/(m^2^ s Pa).

## Supplementary Materials

The following are available online at https://www.mdpi.com/2073-4360/13/6/931/s1, Figure S1: Permeability vs. permselectivity for tBTpCl-APAF, HPA, membranes, and MMMs containing PPN-1 and PPN-2 fillers before and after thermal rearrangement for the O_2_/N_2_ (left) and CO_2_/CH_4_ (right) gas pairs, Table S1: Acronyms list for the polymers and membranes manufactured, Table S2: Permeability coefficients (Barrer) at 3 bar (300 kPa) and 35 °C for HPA-MMMs and their corresponding TR-MMM-HPAs with loads of 20% of PPN-1 and PPN-2, Figure S2: NMR results for membrane HPA, Figure S3: NMR results for membrane PA, Figure S4: NMR results for membrane HPA-PA, Figure S5. TGA thermograms for: PA and MMM-PA (A), HPA-PA (B), and MMM-HPA-PA (D). Samples were heated from 50 to 800 °C at 5 °C/min under a N_2_ atmosphere.

## References

[1] Kim J.S., Moon S.J., Wang H.H., Kim S., Lee Y.M. (2019). Mixed matrix membranes with a thermally rearranged polymer and ZIF-8 for hydrogen separation. J. Membr. Sci..

[2] Al-Mufachi N., Rees N., Steinberger-Wilkens R. (2015). Hydrogen selective membranes: A review of palladium-based dense metal membranes. Renew. Sustain. Energy Rev..

[3] IEA The Future of Hydrogen. https://www.iea.org/reports/the-future-of-hydrogen.

[4] Glenk G., Reichelstein S. (2019). Economics of converting renewable power to hydrogen. Nat. Energy.

[5] Kim S., Shamsaei E., Lin X., Hu Y., Simon G.P., Seong J.G., Kim J.S., Lee W.H., Lee Y.M., Wang H. (2018). The enhanced hydrogen separation performance of mixed matrix membranes by incorporation of two-dimensional ZIF-L into polyimide containing hydroxyl group. J. Membr. Sci..

[6] Luo S., Zhang Q., Zhu L., Lin H., Kazanowska B.A., Doherty C.M., Hill A.J., Gao P., Guo R. (2018). Highly Selective and Permeable Microporous Polymer Membranes for Hydrogen Purification and CO_2_ Removal from Natural Gas. Chem. Mater..

[7] Bakonyi P., Kumar G., Nemestóthy N., Lin C., Bélafi-Bakó K. (2013). Biohydrogen purification using a commercial polyimide membrane module: Studying the effects of some process variables. Int. J. Hydrogen Energy.

[8] Kumar G., Bakonyi P., Kobayashi T., Xu K.-Q., Sivagurunathan P., Kim S.-H., Buitrón G., Nemestóthy N., Bélafi-Bakó K. (2016). Enhancement of biofuel production via microbial augmentation: The case of dark fermentative hydrogen. Renew. Sustain. Energy Rev..

[9] Schorer L., Schmitz S., Weber A. (2019). Membrane based purification of hydrogen system (MEMPHYS). Int. J. Hydrog. Energy.

[10] Li P., Wang Z., Qiao Z., Liu Y., Cao X., Li W., Wang J., Wang S. (2015). Recent developments in membranes for efficient hydrogen purification. J. Membr. Sci..

[11] Dechnik J., Gascon J., Doonan C.J., Janiak C., Sumby C.J. (2017). Mixed-Matrix Membranes. Angew. Chem. Int. Ed..

[12] Vinoba M., Bhagiyalakshmi M., Alqaheem Y., Alomair A.A., Pérez A., Rana M.S. (2017). Recent progress of fillers in mixed matrix membranes for CO2 separation: A review. Sep. Purif. Technol..

[13] Chung T.-S., Jiang L.Y., Li Y., Kulprathipanja S. (2007). Mixed matrix membranes (MMMs) comprising organic polymers with dispersed inorganic fillers for gas separation. Prog. Polym. Sci..

[14] Etxeberria-Benavides M., David O., Johnson T., Łozińska M.M., Orsi A., Wright P.A., Mastel S., Hillenbrand R., Kapteijn F., Gascon J. (2018). High performance mixed matrix membranes (MMMs) composed of ZIF-94 filler and 6FDA-DAM polymer. J. Membr. Sci..

[15] Li X., Jiang Z., Wu Y., Zhang H., Cheng Y., Guo R., Wu H. (2015). High-performance composite membranes incorporated with carboxylic acid nanogels for CO2 separation. J. Membr. Sci..

[16] Smith S.J., Hou R., Lau C.H., Konstas K., Kitchin M., Dong G., Lee J., Lee W.H., Seong J.G., Lee Y.M. (2019). Highly permeable Thermally Rearranged Mixed Matrix Membranes (TR-MMM). J. Membr. Sci..

[17] Soto C., Lugo C.A., Rodríguez S., Palacio L., Lozano Á.E., Prádanos P., Hernandez A. (2020). Enhancement of CO_2_/CH_4_ permselectivity via thermal rearrangement of mixed matrix membranes made from an o-hydroxy polyamide with an optimal load of a porous polymer network. Sep. Purif. Technol..

[18] Han S.H., Kwon H.J., Kim K.Y., Seong J.G., Park C.H., Kim S., Doherty C.M., Thornton A.W., Hill A.J., Lozano Á.E. (2012). Tuning microcavities in thermally rearranged polymer membranes for CO2 capture. Phys. Chem. Chem. Phys..

[19] Cheng Y., Ying Y., Japip S., Jiang S.-D., Chung T.-S., Zhang S., Zhao D. (2018). Advanced Porous Materials in Mixed Matrix Membranes. Adv. Mater..

[20] Lin R., Hernandez B.V., Ge L., Zhu Z. (2018). Metal organic framework based mixed matrix membranes: An overview on filler/polymer interfaces. J. Mater. Chem. A.

[21] Ben T., Ren H., Ma S., Cao D., Lan J., Jing X., Wang W., Xu J., Deng F., Simmons J.M. (2009). Targeted Synthesis of a Porous Aromatic Framework with High Stability and Exceptionally High Surface Area. Angew. Chem. Int. Ed..

[22] Lau C.H., Mulet X., Konstas K., Doherty C.M., Sani M.-A., Separovic F., Hill M.R., Wood C.D. (2016). Hypercrosslinked Additives for Ageless Gas-Separation Membranes. Angew. Chem. Int. Ed..

[23] Aguilar-Lugo C., Suárez-García F., Hernández A., Miguel J.A., Lozano Á.E., de la Campa J.G., Álvarez C. (2019). New Materials for Gas Separation Applications: Mixed Matrix Membranes Made from Linear Polyimides and Porous Polymer Networks Having Lactam Groups. Ind. Eng. Chem. Res..

[24] Lopez-Iglesias B., Suárez-García F., Aguilar-Lugo C., Ortega A.G., Bartolomé C., Martínez-Ilarduya J.M., de la Campa J.G., Lozano Á.E., Álvarez C. (2018). Microporous Polymer Networks for Carbon Capture Applications. ACS Appl. Mater. Interfaces.

[25] García C., Lozano A.E., de la Campa J.G., de Abajo J. (2003). Soluble Polyimides from a New Dianhydride: 5′-tert-Butyl-m-terphenyl-3,4,3′′,4′′-tetracarboxylic Acid Dianhydride. Macromol. Rapid Commun..

[26] García C., Tiemblo P., Lozano A.E., de Abajo J., de la Campa J.G. (2002). Gas separation properties of new poly(aryl ether ketone)s with pendant groups. J. Membr. Sci..

[27] Bermejo L.A., Alvarez C., Maya E.M., García C., de la Campa J.G., Lozano A.E. (2018). Synthesis, characterization and gas separation properties of novel polyimides containing cardo and tert-butyl-m-terphenyl moieties. Express Polym. Lett..

[28] de Abajo J., de la Campa J.G., Lozano A.E. (2003). Designing aromatic polyamides and polyimides for gas separation membranes. Macromol. Symp..

[29] Klotz E.J.F., Claridge T.D.W., Anderson H.L. (2006). Homo- and Hetero-[3]Rotaxanes with Two π-Systems Clasped in a Single Macrocycle. J. Am. Chem. Soc..

[30] Smith Z.P., Czenkusch K., Wi S., Gleason K.L., Hernández G., Doherty C.M., Konstas K., Bastow T.J., Álvarez C., Hill A.J. (2014). Investigation of the chemical and morphological structure of thermally rearranged polymers. Polymer.

[31] Miyaura N., Suzuki A. (1995). Palladium-Catalyzed Cross-Coupling Reactions of Organoboron Compounds. Chem. Rev..

[32] Liao D.C., Hsieh K.H. (1994). Synthesis and characterization of bismaleimides derived from polyurethanes. J. Polym. Sci. Part A Polym. Chem..

[33] Smith Z.P., Sanders D.F., Ribeiro C.P., Guo R., Freeman B.D., Paul D.R., McGrath J.E., Swinnea S. (2012). Gas sorption and characterization of thermally rearranged polyimides based on 3,3′-dihydroxy-4,4′-diamino-biphenyl (HAB) and 2,2′-bis-(3,4-dicarboxyphenyl) hexafluoropropane dianhydride (6FDA). J. Membr. Sci..

[34] Muñoz D.M., Calle M., de la Campa J.G., de Abajo J., Lozano A.E. (2009). An Improved Method for Preparing Very High Molecular Weight Polyimides. Macromolecules.

[35] Muñoz D.M., de la Campa J.G., de Abajo J., Lozano A.E. (2007). Experimental and Theoretical Study of an Improved Activated Polycondensation Method for Aromatic Polyimides. Macromolecules.

[36] Lozano A.E., de Abajo J., de la Campa J.G. (1997). Synthesis of Aromatic Polyisophthalamides by in Situ Silylation of Aromatic Diamines. Macromolecules.

[37] Lozano A.E., de Abajo J., De la Campa J.G. (1998). Quantum semiempirical study on the reactivity of silylated diamines in the synthesis of aromatic polyamides. Macromol. Theory Simul..

[38] Comesaña-Gándara B., Calle M., Jo H.J., Hernández A., de la Campa J.G., de Abajo J., Lozano A.E., Lee Y.M. (2014). Thermally rearranged polybenzoxazoles membranes with biphenyl moieties: Monomer isomeric effect. J. Membr. Sci..

[39] Sanders D.F., Smith Z.P., Ribeiro C.P., Guo R., McGrath J.E., Paul D.R., Freeman B.D. (2012). Gas permeability, diffusivity, and free volume of thermally rearranged polymers based on 3,3′-dihydroxy-4,4′-diamino-biphenyl (HAB) and 2,2′-bis-(3,4-dicarboxyphenyl) hexafluoropropane dianhydride (6FDA). J. Membr. Sci..

[40] Wiederhorn S., Fields R., Low S., Bahng G.-W., Wehrstedt A., Hahn J., Tomota Y., Miyata T., Lin H., Freeman B., Czichos H., Saito T., Smith L. (2006). Mechanical Properties. Handbook of Materials Measurement Methods.

[41] Smith Z.P., Hernández G., Gleason K.L., Anand A., Doherty C.M., Konstas K., Alvarez C., Hill A.J., Lozano A.E., Paul D.R. (2015). Effect of polymer structure on gas transport properties of selected aromatic polyimides, polyamides and TR polymers. J. Membr. Sci..

[42] Díez B., Cuadrado P., Marcos-Fernández Á., de la Campa J.G., Tena A., Prádanos P., Palacio L., Lee Y.M., Alvarez C., Lozano Á.E. (2018). Thermally rearranged polybenzoxazoles made from poly(ortho-hydroxyamide)s. Characterization and evaluation as gas separation membranes. React. Funct. Polym..

[43] Wang H., Chung T.-S., Paul D. (2014). Thickness dependent thermal rearrangement of an ortho-functional polyimide. J. Membr. Sci..

[44] Park H.B., Jung C.H., Lee Y.M., Hill A.J., Pas S.J., Mudie S.T., Van Wagner E., Freeman B.D., Cookson D.J. (2007). Polymers with Cavities Tuned for Fast Selective Transport of Small Molecules and Ions. Science.

[45] Galizia M., Chi W.S., Smith Z.P., Merkel T.C., Baker R.W., Freeman B.D. (2017). 50th Anniversary Perspective: Polymers and Mixed Matrix Membranes for Gas and Vapor Separation: A Review and Prospective Opportunities. Macromolecules.

[46] Park H.B., Han S.H., Jung C.H., Lee Y.M., Hill A.J. (2010). Thermally rearranged (TR) polymer membranes for CO2 separation. J. Membr. Sci..

[47] Rezakazemi M., Amooghin A.E., Montazer-Rahmati M.M., Ismail A.F., Matsuura T. (2014). State-of-the-art membrane based CO_2_ separation using mixed matrix membranes (MMMs): An overview on current status and future directions. Prog. Polym. Sci..

[48] Robeson L.M. (1991). Correlation of separation factor versus permeability for polymeric membranes. J. Membr. Sci..

[49] Robeson L.M. (2008). The upper bound revisited. J. Membr. Sci..

[50] Robeson L., Burgoyne W., Langsam M., Savoca A., Tien C. (1994). High performance polymers for membrane separation. Polymer.

[51] Brunetti A., Cersosimo M., Kim J.S., Dong G., Fontananova E., Lee Y.M., Drioli E., Barbieri G. (2017). Thermally rearranged mixed matrix membranes for CO 2 separation: An aging study. Int. J. Greenh. Gas Control..

[52] Thornton A.W., Nairn K.M., Hill A.J., Hill J.M. (2009). New relation between diffusion and free volume: I. Predicting gas diffusion. J. Membr. Sci..

[53] Matteucci S., Yampolskii Y., Freeman B.D., Pinnau I., Yampolskii Y., Pinnau I., Freeman B.D. (2006). Transport of Gases and Vapors in Glassy and Rubbery Polymers. Materials Science of Membranes for Gas and Vapor Separation.

[54] Teplyakov V.V., Durgar’yan S.G. (1984). Correlation analysis of the gas permeability parameters of polymers. Polym. Sci. USSR.

[55] Breck D.W. (1974). Zeolite Molecular Sieves: Structure, Chemistry and Use.

