# Supplementary material for: Gas Separation by Mixed Matrix Membranes with Porous Organic Polymer Inclusions within o-Hydroxypolyamides Containing m-Terphenyl Moieties
Source: Polymers (Basel). 2021 Mar 18;13(6):931. doi: 10.3390/polym13060931 (PMC8003052; doi:10.3390/polym13060931)
Supplement: Supplementary file 1 [file polymers-13-00931-s001.pdf]

Gas separation by mixed matrix membranes with porous organic polymer inclusions within o-hydroxypolyamides containing m-terphenyl moieties.

Cenit Soto[1,2], Edwin S. Torres Cuevas[3], Alfonso González-Ortega[4], Laura Palacio[1,2], Ángel E. Lozano[1,5,6], Benny D. Freeman[3], Pedro. Prádanos[1,2,*], Antonio. Hernandez[1,2,*].

[1] Surfaces and Porous Materials (SMAP), Associated Research Unit to CSIC. University of Valladolid, Facultad de Ciencias, Paseo Belén 7, E-47011 Valladolid, Spain.
[2] Institute of Sustainable Processes (ISP), Dr. Mergelina s/n, 47011, Valladolid, Spain.
[3] McKetta Department of Chemical Engineering, Texas Materials Institute, The University of Texas at Austin
[4] Department of Organic Chemistry, School of Sciences, University of Valladolid, Facultad de Ciencias, Paseo Belén 7, E-47011 Valladolid, Spain.
[5] Institute for Polymer Science and Technology (ICTP-CSIC), Juan de la Cierva 3, 28006 Madrid, Spain
[6] IU CINQUIMA, University of Valladolid, Paseo Belén 5, E-47011 Valladolid, Spain.

*Correspondence: antonio.hernandez@uva.es (A.H.); ppradanos@uva.es (P.P.)

Supplementary materials

SI-1. Polymers and Membranes Manufactured

Table S1. Acronyms list for the polymers and membranes manufactured.

**Polymers (polymeric matrix)**	**Acronym**
5'-tertbutyl-*m*-terphenyl-4,4''-dichloride acid (tBTpCl) + 2,2-bis(3-amino-4-hydroxy phenyl)-hexafluoropropane (APAF). → **tBTpCl-APAF**	**HPA**
5'-tertbutyl-*m*-terphenyl-4,4''-dichloride acid (tBTpCl) + 4,4′-(hexafluoroisopropylidene) dianiline (6FpDA). → **tBTpCl-6FpDA**	**PA**
5'-tertbutyl-*m*-terphenyl-4,4''-dichloride acid (tBTpCl) + 2,2-bis(3-amino-4-hydroxy phenyl)-hexafluoropropane (APAF) + 4,4′-(hexafluoroisopropylidene) dianiline (6FpDA). → **tBTpCl-APAF-6FpDA**	**HPA-PA**
**Mixed Matrix Membranes**	
tBTpCl-APAF + 20% PPN-2	**MMM-HPA**
tBTpCl-6FpDA + 20% PPN-2	**MMM-PA**
tBTpCl-APAF-6FpDA + 20% PPN-2	**MMM-HPA-PA**

Thermal rearrangement tBTpCl-APAF + 20% PPN-2	**TR-MMM-HPA**
Thermal rearrangement tBTpCl-APAF-6FpDA + 20% PPN-2	**TR-MMM-HPA-PA**

SI-2. Preliminary gas separation properties

In a preliminary study, and to assess the influence of this filler on gas transport properties (especially for CO_2 separation), gas permeation measurements were performed at 35 °C and 3 bars. The comparative evaluation of gas permeabilities for MMMs and TR-MMMs with the two tested PPNs are shown in Table S2. These results denoted a pronounced difference of permeabilities between the fillers, in particular for TR-MMMs. Thus, an enhancement factor of at least 5-fold was recorded for the permeability of CO_2 for TR-MMMs from PPN-2, which reach ~394 Barrer but with a rather lower selectivity. Despite the fact that isatin-derived PPN-1 have narrower micropores, [24], this empirical findings showed that PPN-2 is much better for making high-performance MMMs, and consequently PPN-1 was discharged for additional characterization. A plausible explanation to this fact could explained by the different interactions of the filler with the polymer matrix [16].

Table S2. Permeability Coefficients (Barrer) at 3 bar (300 kPa) and 35 °C for HPA-MMMs and their corresponding TR-MMM-HPAs with loads of 20% of PPN-1 and PPN-2.

**Membrane**	**Permeability (Barrer*)**			
	N_2	O_2	CH_4	CO_2
	**PPN-1**			
**tBTpCl-APAF-20%, MMM-HPA-PPN-1**	1.03	5.98	0.67	24.85
**TR-tBTpCl-APAF-20% TR-MMM-HPA-PPN-1**	13.38	55.10	11.99	243.4
	**PPN-2**			
**tBTpCl-APAF-20% MMM-HPA-PPN-2**	3.48	18.46	2.65	79.0
**TR-tBTpCl-APAF-20% TR-MMM-HPA-PPN-2**	20.65	87.97	20.80	394.1

Figure S1 shows the correlation for O_2/N_2 and CO_2/CH_4 and in the Robeson's limit for the tested MMMs with PPN-1 and PPN-2 and their corresponding TR-MMM. Note

that, despite PPN-1 synthesized from triptycene-isatin exhibited a higher CO_2 capture (207 mg g^{-1}) than PPN-2 synthesized from triptycene-TFAP (83 mg g^{-1}) [24] the permeabilities of all measured gases were lower for the MMMs derived form PPN-1. This could be attributed to a low interaction between the filler and the polymer matrix, which consequently would result in a poor adhesion and the formation of interfacial voids that were not fixed during thermal rearrangement this should explain as well the lower selectivity for the MMMs containing PPN-2. In any case the different behavior of both PPNs lead to a better permeability-selectivity compromise as seen in Figure S1.

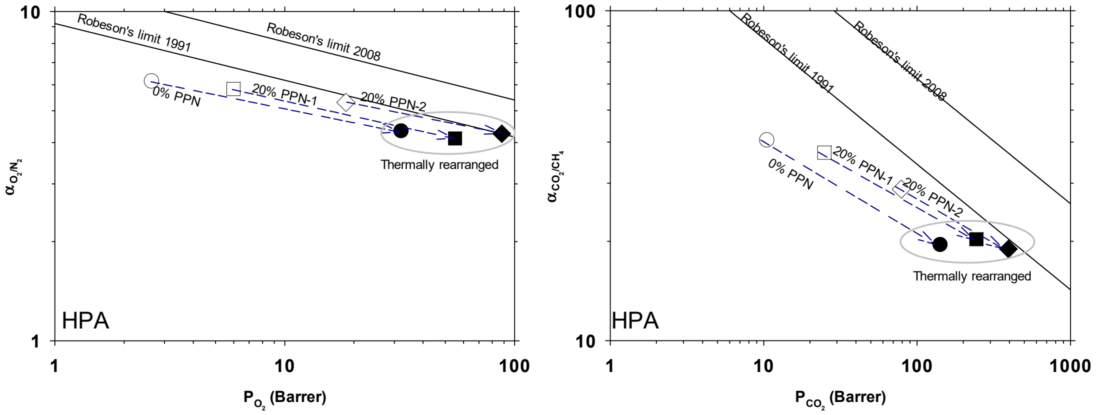

Figure S1. Permeability vs permselectivity for tBTpCl-APAF, HPA, membranes and MMMs containing PPN-1 and PPN-2 fillers before and after thermal rearrangement for the O_2/N_2 (left) and CO_2/CH_4 (right) gas pairs.

SI-3. NMR characterization of polymers

tBTpCl-APAF (**HPA**) → 5'-terbutyl-*m*-terphenyl-4,4'' acid dichloride + 2,2-bis(3-amino-4-hydroxyphenyl)-hexafluoropropane

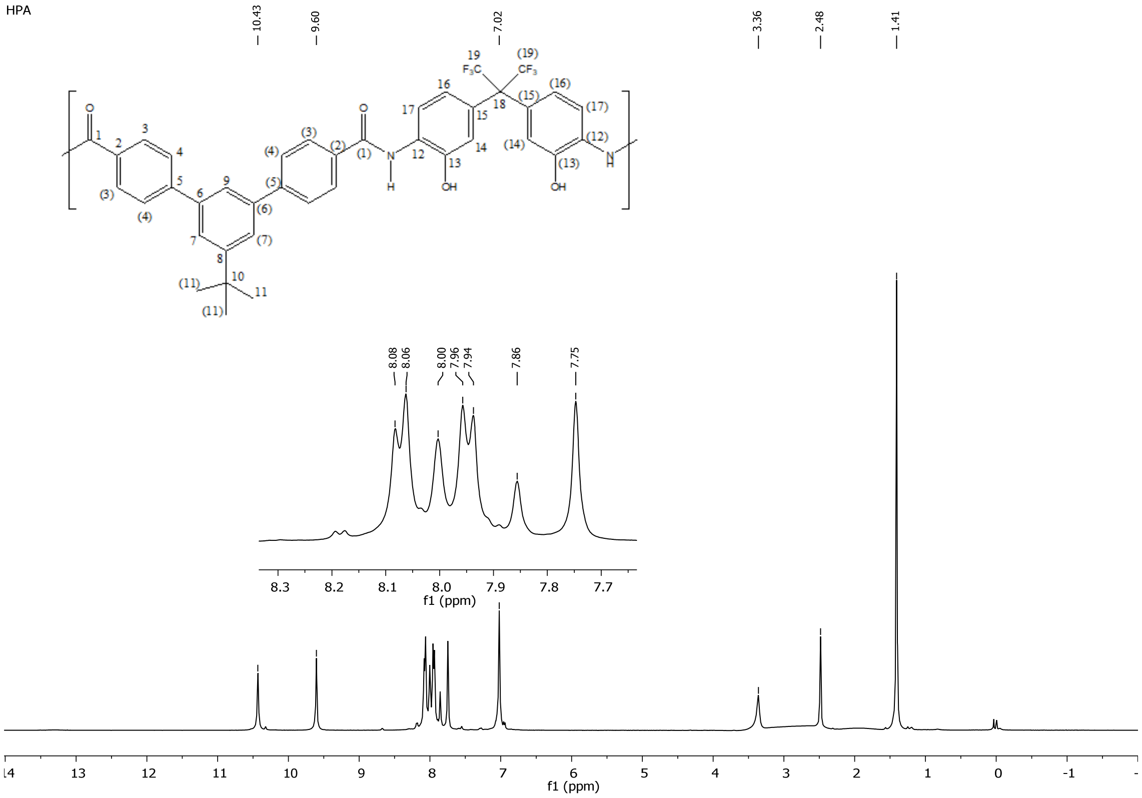

^{1}H-RMN (400 MHz, DMS d_6: δ (ppm); 10.43 (2 *NH*), 9.60 (2 *OH*), 8.07 (4 *H(3)*), 8.0 (2 H(14)), 7.95 (4 $H_{(4)}$), 7.86 (1 $H_{(9)}$), 7.75 (2 $H_{(7)}$), 7.02 (2 $H_{(17)}$) , 2 $H_{(16)}$), 3.36 (H_2O), 2.48 (DMS), 1.41 (9 $H_{(11)}$).

Figure S2. NMR results for membrane HPA.

tBTpCl-6FpDA (**PA**) → 5'-terbutyl-*m*-terphenyl-4,4'' acid dichloride + 2,2-bis(4-aminophenyl)hexafluoropropane.

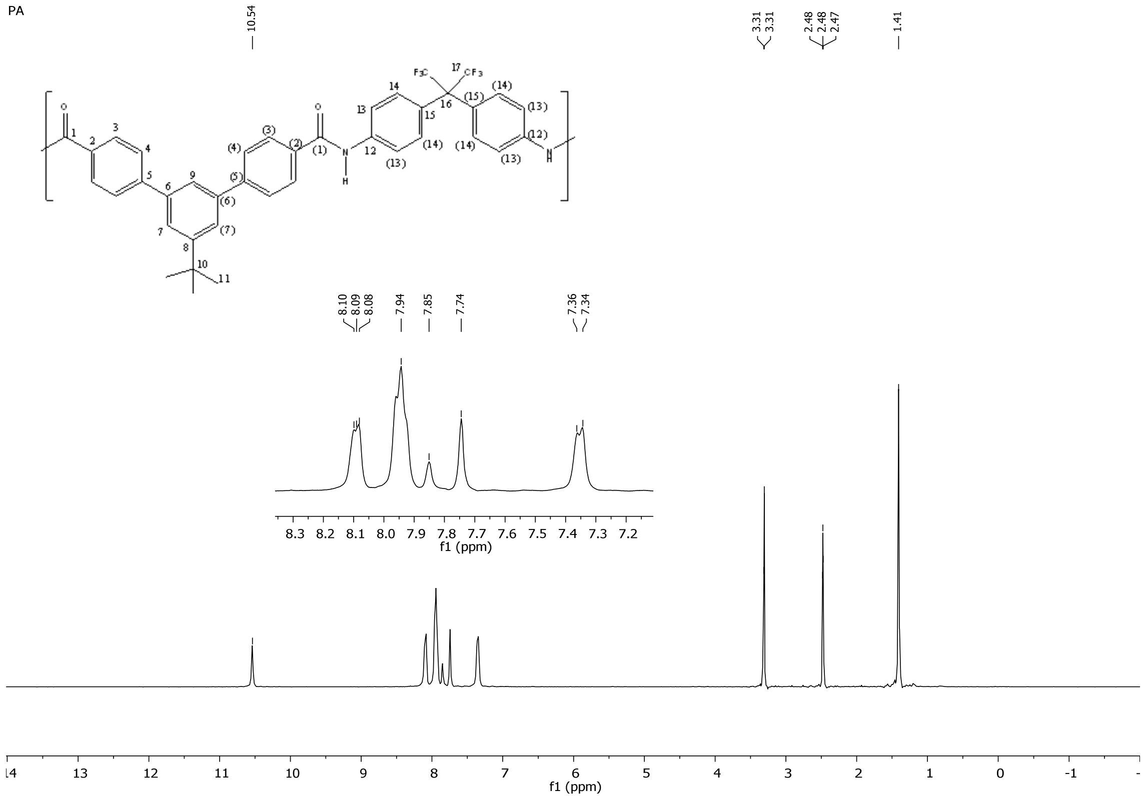

^{1}H-RMN (400 MHz, DMS d_6: δ (ppm); 10.54 (2 *NH*), 8.09 (4 H(3)), 7.94 (4 $H_{(4)}$, 4 $H_{(13)}$), 7.85 (1 $H_{(9)}$), 7.74 (2 $H_{(7)}$), 7.75 (2 $H_{(7)}$), 7.35 (2 $H_{(17)}$, 4 $H_{(14)}$), 3.31 (H_2O), 2.48 (DMSO), 1.41 (9 $H_{(11)}$).

Figure S3.- NMR results for membrane PA.

tBTpCl-APAF-6FpDA (**HPA-PA**) → 5'-terbutyl-*m*-terphenyl-4,4'' acid dichloride **+** 2,2-bis(3-amino-4-hydroxyphenyl)-hexafluoropropane + 2,2-bis(4-aminophenyl)hexafluoropropane.

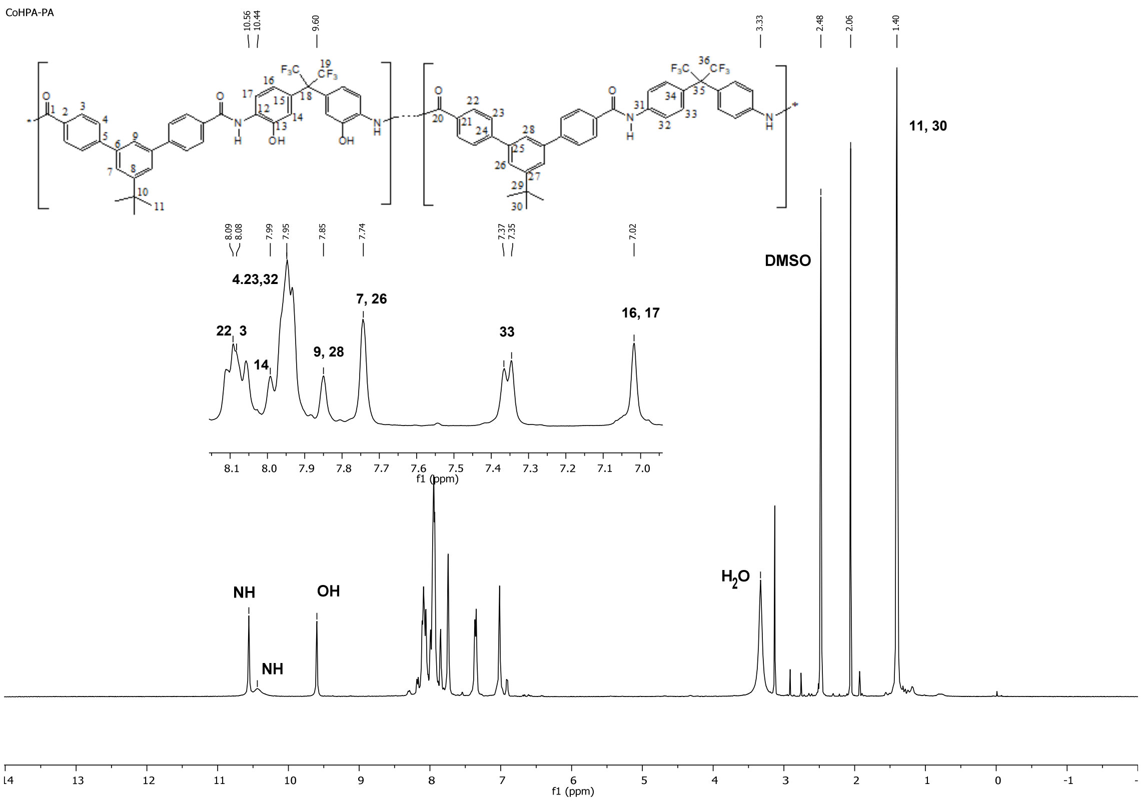

^{1}H-RMN (400 MHz, DMS d_6: δ (ppm) 10.56 (*NH*), 10.44 (NH), 9.60 (OH), 8.09 (H$_{(22)}$, 8.08 (H$_{(3)}$), 7.99 (H$_{(14)}$), 7.95 (H$_{(4,23,32)}$), 7.85 (H$_{(9,28)}$), 7.74 (H$_{(7,26)}$), 7.36 (H$_{(33)}$), 7.02 (H$_{(16,17)}$), 3.33 (H_2O), 2.48 (DMSO), 1.40 (H$_{(11,30)}$).

Figure S4. NMR results for membrane HPA-PA.

SI-4. TGA results.

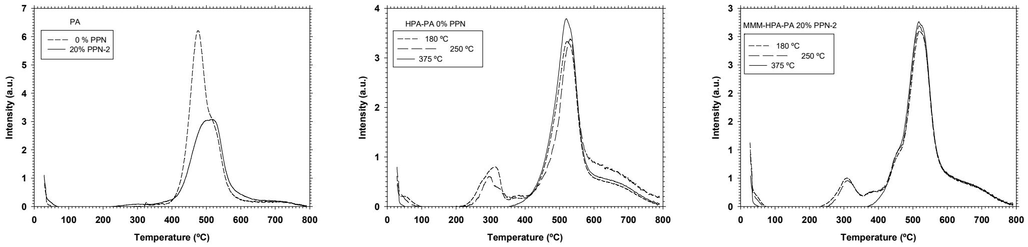

Figure S5. TGA thermograms for: PA and MMM-PA (A), HPA-PA (B) and MMM-HPA-PA (C). Samples were heated from 50 to 800 °C at 5 °C/min under a N_2 atmosphere.
